# Experience of Medical Disputes, Medical Disturbances, Verbal and Physical Violence, and Burnout Among Physicians in China

**DOI:** 10.3389/fpsyg.2020.556517

**Published:** 2021-01-29

**Authors:** Yinuo Wu, Feng Jiang, Jing Ma, Yi-Lang Tang, Mingxiao Wang, Yuanli Liu

**Affiliations:** ^1^Chinese Academy of Medical Sciences and Peking Union Medical College, Beijing, China; ^2^Institute of Health Yangtze River Delta, Shanghai Jiao Tong University, Shanghai, China; ^3^Department of Population Medicine, Harvard Medical School, Boston, MA, United States; ^4^Department of Psychiatry and Behavioral Sciences, Emory University, Atlanta, GA, United States; ^5^Atlanta VA Medical Center, Decatur, GA, United States; ^6^Department of Cardiology, Emergency General Hospital, Beijing, China; ^7^School of Health Policy and Management, Chinese Academy of Medical Sciences and Peking Union Medical College, Beijing, China

**Keywords:** physician burnout, medical disputes, workplace violence, hospitals, China

## Abstract

**Background:**

Medical disputes, medical disturbances, verbal and physical violence against physicians, and burnout have reached epidemic levels. They may negatively impact both physicians and the healthcare system. The experience of medical disputes, medical disturbances, verbal, and physical violence, and burnout and the correlates in physicians working in public hospitals in China needed to be investigated.

**Methods:**

A nationwide cross-sectional survey study was conducted between 18 and 31 March 2019. An anonymous online questionnaire was administered. The questionnaire included the 22-item Maslach Burnout Inventory-Human Services Survey (Chinese version). We also collected data on demographic and job-related factors, as well as physicians’ experiences of medical disputes, medical disturbances, verbal and physical violence from patients and the patients’ family members.

**Findings:**

In total, 22,213 physicians from 144 tertiary public hospitals in all of China’s 31 provinces completed the survey. The overall burnout rate among the surveyed physicians was 31.28%. Moreover, 33.48% of physicians experienced disputes, 20.86% experienced disturbances, 48.52% experienced verbal violence, and 5.84% experienced physical violence in the past 12 months. Factors found to be significantly associated with burnout included younger age, being divorced or widowed, having a lower educational background, working in internal medicine departments, longer working hours per day, working in general hospitals, being in East China, as well as having experienced disputes, disturbances, and physical and verbal violence.

**Interpretation:**

Close to a third of the Chinese doctors working in the tertiary hospitals reportedly experienced burnout, and the problem is related to the unsafe working environment caused by the worsening doctor-patient relationship.

## Introduction

Burnout is a work-related condition with symptoms consisting of emotional exhaustion, depersonalization, and a sense of reduced personal accomplishment (West et al., 2018). Physician burnout has been linked to adverse medical care, medical errors, poor physicians’ health and safety, including a higher risk of depression, alcohol abuse/dependence, and suicidal ideation, and higher intention to leave (Shanafelt et al., 2016; Collier, 2017; Panagioti et al., 2018; West et al., 2018; Dyer, 2019; Hu et al., 2019). It may also negatively impact healthcare organizations and systems. Several studies, involving different settings and specialties, showed that the prevalence of burnout was around 50% in practicing physicians (Embriaco et al., 2007; Rotenstein et al., 2018; Gas et al., 2019; Lin et al., 2019; Ma et al., 2019; The Lancet, 2019). Existing Chinese studies showed that the prevalence of burnout among oncologists, neurologists, and intensivists in China ranged from 51% to 61% (Zhou et al., 2017; See et al., 2018; Ma et al., 2019).

Negative workplace experiences, including disputes, disturbances, and verbal and physical violence, can create an unsafe and hostile working environment and lead to physician burnout (Hu et al., 2019). Medical disputes refer to any disputes between doctors (hospitals) and patients (and/or their family members) related to diagnosis and treatments (State Council of the PRC, 2018). A purposeful disturbance (*Yi Nao* in Chinese) is a type of medical dispute in which patients, their family members, or even “hired individuals” attempt to seek compensation for actual or perceived malpractice or dissatisfaction with services (such as death, unsuccessful surgeries, adverse side effects of medication, etc.). In doing so, they often threaten violence or assault medical staff (Hesketh et al., 2012; Zhou and Hao, 2019). This financially motivated mistreatment and violence against physicians is particularly common and severe in China, although the reasons are complex (Guo et al., 2019; The Lancet, 2020). Those medical mistreatments have further worsened the working environment for physicians, which may harm health care services. According to two previous surveys, the prevalences of disputes, verbal and physical violence reported by Chinese physicians were 7.96, 75.2, and 5.4%, respectively (Lu et al., 2015; Guo et al., 2019).

Despite frequent media reports of doctor-patient disputes and patient-led verbal and physical violence against physicians in China, there has been a gap in the literature on a comprehensive understanding of China’s physician burnout and its association with the rising incidence of disputes and violence in the Chinese healthcare facilities. A few previous estimates of mistreatment and burnout in physicians were based on surveys with relatively small sample size or involved only one specialty or a limited number of local hospitals (Li et al., 2003; Bennett et al., 2005; Chen et al., 2008; Fan et al., 2012; Kumar et al., 2013; Liang et al., 2014; Lin and Li, 2015; Kealy et al., 2016; Banerjee et al., 2017; Busis et al., 2017; Rassolian et al., 2017; Balzora and Weinshel, 2018; Brashear and Vickrey, 2018; Burki, 2018; Chang et al., 2018; Patti et al., 2018; Hu et al., 2019; Ma et al., 2019). One study conducted among surgery residents in the United States found that burnout was associated with verbal or physical abuse (Hu et al., 2019). It is essential to study the frequency and extent of burnout and mistreatment involving multiple hospitals and all typical clinical specialties. This survey was set to collect such data and examine the associations between physician burnout and demographic and job-related factors, especially mistreatment experiences.

## Data and Methods

### Study Design and Samples

The study was a part of the China National Healthcare Improvement Initiative Survey in 2019 (Zhou et al., 2018). The National Health Commission of China approved and supported this project. The survey was conducted on March 18–31, 2019. Totally 144 tertiary public hospitals in capital cities of the 31 provinces in mainland China were selected to participate in the survey. Among them were 59 general hospitals, 37 Traditional Chinese Medicine (TCM) hospitals, 33 maternal and children’s hospitals, five stomatological hospitals, four cancer hospitals, and six other specialty hospitals.

Based on their employee’s ID codes in the hospital staff lists, physicians were sampled through a systematic sampling method in each participating hospital. We invited 170 physicians from each hospital to participate in the survey. The survey was conducted anonymously through WeChat, a popular online social media application in China.

### Ethics Statement

The protocol of this study was approved by the Ethics Committee (IEC) of the Emergency General Hospital in Beijing, China. All participants signed the informed consent before they proceeded to respond to the questionnaires. The informed consent statement explained the purpose of the survey, ensured that the data would be de-identified before analysis and that the hospitals’ administrators would not have access to their responses.

### Measures

The items on medical disputes, purposeful disturbances, and violence were adapted from previously published and validated instruments (Liang et al., 2014; Lu et al., 2015; Guo et al., 2019; Zhou and Hao, 2019). Detailed operational definitions for medical disputes, medical disturbances, and violence were included in the questionnaire for better reliability.

Respondents were asked to report whether they experienced medical disputes or medical disturbances and reported the frequency of verbal violence and physical violence from patients and/or patients’ family members in the previous year.

The dimensions of burnout were assessed using the Maslach Burnout Inventory–Human Services Survey (MBI–HSS) (Chinese version), which was developed by the Chinese Academy of Sciences with solid reliability and validity (Li et al., 2003). It includes three subscales: emotional exhaustion (EE), depersonalization (DP), and low personal accomplishment (PA). As there are different thresholds for burnout (Rotenstein et al., 2018), we defined EE as EE score ≥27, DP as DP score ≥10, and PA as PA score ≤33. The overall burnout was defined as EE score ≥27 and/or DP score ≥10 (Maslach et al., 2010).

Additionally, we collected participants’ sociodemographic data, including age, sex, marital status, number of children, educational level; and job-related factors, including their departments, working hours per day, hospital type and the regions where the participants worked (East China, Middle China, and West China).

Before the survey was made available to all participants, a pilot survey was conducted among 150 physicians to assess the questionnaire’s overall coherence, balance, and clarity.

### Statistical Analysis

Descriptive analyses were conducted for the variables. Continuous variables were shown with mean and standard deviation, while categorical variables were shown with percentages. Age and working hours were treated as a continuous variable. Chi-square tests were used to examine all categorical variables. Multivariable logistic regression models were used to examine all variables associated with burnout. Models of excluding and including mistreatment exposure were used to test the effects of mistreatment on burnout. The primary model examined the association between the composite mistreatment variable and burnout. All models were estimated with robust standard errors as physicians clustering within hospitals. Missing data were rare (<1%) and were excluded from the analyses.

Stata 15 (StataCorpLP, College Station, TX, United States) was used for these statistical analyses. All of the tests were two-sided, and the statistical significance was defined as *P* < 0.05.

## Results

### Description of Sample Characteristics and Related Factors

In total, 24,480 physicians were invited to participate, and 22,416 responded (response rate = 91.57%). After removing 203 participants with incomplete data, data from 22,213 physicians (99.09%) were included in the analysis. Their sociodemographic characteristics are shown in Table 1.

**TABLE 1:** Characteristics of physicians in tertiary public hospitals (*N* = 22,213).

Characteristic	Number	Percent (%)
**Sex**		
Male	9415	42.39
Female	12798	57.61
**Marriage status**		
Single	3251	14.64
Married	18335	82.54
Divorced or widowed	627	2.82
**Children**		
None	5832	26.25
One	12840	57.80
More than one	3541	15.94
Educational level*		
Bachelor degree or below	5657	25.47
Master’s degree	10467	47.12
Doctorate degree	6089	27.41
**Department**		
Internal Medicine	6607	29.74
Surgery	7305	32.89
Ob/Gyn**	3107	13.99
Pediatrics	2236	10.07
Emergency	1031	4.64
Others***	1927	8.68
**Hospital type**		
General hospitals	8900	40.07
TCM general hospitals	6126	27.58
Specialty Hospitals	7187	32.35
**Location**		
East China	9584	43.15
Central China	5268	23.72
West China	7361	33.14
Age (Mean ± SD)	37.97 ± 8.15	
Working Hours/day (Mean ± SD)	9.62 ± 1.93	

*In China, medical school graduates are awarded with a bachelor degree of medicine (similar to the European and Russian systems). Some obtained a master’s or doctorate degree in addition to their medical degree. **Ob/Gyn: obstetrics-gynecology. ***Including oncology department, rehabilitation department, reproductive department, geriatrics department, etc.

Among all participants, 57.61% were women, 82.54% were married, and nearly three quarters (73.75%) had at least one child, and a similar percentage (74.53%) had a master’s or a doctorate degree in addition to an undergraduate medical degree. More than one third were in the surgery department (surgeons), 40.07% worked in general hospitals, and 43.15% worked in East China. The mean age was 37.97 ± 8.15 years old. On average, they worked 9.62 ± 1.93 h per day, and 66.54% worked longer than 8 h per day, exceeding the limit set by the Labor Law of China (China Legal System Publishing House, 2018).

### Burnout Among Physicians

Table 2 shows the distribution of burnout across different subgroups of physicians. Overall burnout was reported by 31.28% physicians, with 17.89% reporting symptoms of EE, 27.60% reporting DP and 48.27% reporting PA. As shown in Table 3, physicians who were male, single, without children, working in pediatric departments, in general hospitals, in West China, experienced a higher rate of overall burnout.

**TABLE 2:** Distribution of burnout.

Variable	Overall burnout (%)	
	None	Happened
Sex	*P* = 0.097	
Male	6413 (68.11)	3002 (31.89)
Female	8851 (69.16)	3947 (30.84)
Marital status	*P* < 0.0001	
Single	2063 (63.46)	1188 (36.54)
Married	12783 (69.72)	5552 (30.28)
Divorced or widowed	418 (66.67)	209 (33.33)
Children	*P* < 0.0001	
None	3750 (64.30)	2082 (35.70)
One	9027 (70.30)	3813 (29.70)
More than one	2487 (70.23)	1054 (29.77)
Educational level	*P* < 0.0001	
Bachelor degree or below	3937 (69.60)	1720 (30.40)
Master’s degree	7013 (67.00)	3454 (33.00)
Doctorate degree	4314 (70.85)	1775 (29.15)
Department	*P* < 0.0001	
Internal medicine	4445 (67.28)	2162 (32.72)
Surgery	5116 (70.03)	2189 (29.97)
Ob/Gyn	2138 (68.81)	969 (31.19)
Pediatrics	1426 (63.77)	810 (36.23)
Emergency	744 (72.16)	287 (27.84)
Others	1395 (72.39)	532 (27.61)
Hospital type	*P* < 0.0001	
General hospitals	5895 (66.24)	3005 (33.76)
TCM general hospitals	4307 (70.31)	1819 (29.69)
Specialty hospitals	5062 (70.43)	2125 (29.57)
Location	*P* = 0.0461	
East China	6610 (68.97)	2974 (31.03)
Central China	3671 (69.68)	1597 (30.32)
West China	4983 (67.69)	2378 (32.31)

**TABLE 3:** Factors associated with burnout among Chinese physicians.

Characteristic	Burnout					
	Model excluding maltreatment factors			Model including maltreatment factors		
	Odds ratio	95% CI (lower)	95% CI (upper)	Odds ratio	95% CI (lower)	95% CI (upper)
**Sex**						
Male	Reference					
Female	0.91	0.86	0.97	1.04	0.97	1.11
Age	0.97	0.97	0.98	0.97	0.96	0.97
Marital status						
Single	Reference					
Married	0.98	0.88	1.10	1.04	0.92	1.16
Divorced or widowed	1.28	1.05	1.57	1.31	1.06	1.62
**Children**						
None	Reference					
One	1.03	0.93	1.13	1.01	0.91	1.11
More than one	0.97	0.87	1.09	0.97	0.87	1.10
**Educational level**						
Bachelor degree or below	Reference					
Master’s degree	0.94	0.88	1.02	0.96	0.89	1.04
Doctorate degree	0.81	0.74	0.88	0.84	0.77	0.93
**Department**						
Surgery	Reference					
Internal medicine	1.16	1.08	1.26	1.11	1.03	1.21
Ob/Gyn	1.09	0.99	1.21	0.98	0.88	1.10
Pediatrics	1.35	1.21	1.50	1.05	0.94	1.18
Emergency	1.04	0.89	1.20	1.05	0.89	1.23
Others	0.94	0.84	1.06	0.97	0.86	1.09
Working Hours/day	1.17	1.15	1.19	1.12	1.11	1.14
**Hospital type**						
General hospitals	Reference					
TCM general hospitals	0.85	0.79	0.91	0.90	0.83	0.97
Specialty hospitals	0.85	0.79	0.91	0.93	0.86	1.01
**Location**						
East China	Reference					
Central China	0.93	0.87	1.01	0.92	0.85	0.99
West China	0.95	0.88	1.02	0.92	0.85	0.99
Medical Disputes				1.30	1.21	1.39
Medical Disturbances				1.17	1.08	1.27
**Verbal violence**						
None	Reference					
A few times a year				2.34	2.18	2.51
A few times per month or more frequently				4.52	4.13	4.94
**Physical violence**						
None	Reference					
A few times a year				1.43	1.25	1.64
A few times per month or more frequently	Verbal abuse			1.36	1.03	1.79

### Medical Disputes, Medical Disturbances, Verbal, and Physical Violence

The frequency and distribution of different types of mistreatment are shown in Tables 4, 5.

**TABLE 4:** Frequency of medical disputes, medical disturbances, verbal and physical violence among Chinese physicians.

Variable	Number (total *N* = 22213)	Percent (%)
Experienced medical disputes in previous year	7436	33.48
Experienced medical disturbances in previous year	4633	20.86
**Verbal violence from patients or patients’ families**		
None	11435	51.48
A few times a year	7150	32.19
A few times per month or more	3628	16.33
**Physical violence from patients or their families**		
None	20917	94.17
A few times a year	1050	4.73
A few times per month or more	246	1.11

**TABLE 5:** Distribution of medical disputes, medical disturbance, verbal and physical violence among Chinese.

Variable	Medical disputes		Physicians		Verbal violence number (percent)			Physical violence		
			Medical disturbances							
	Not experienced	Experienced	Not experienced	Experienced	None	A few times a year	A few times per month or more	None	A few times a year	A few times per month or more
**Sex**										
Male	5701 (60.55)	3714 (39.45)	7069 (75.08)	2346 (24.92)	4531 (48.13)	3212 (34.12)	1672 (17.76)	8656 (91.94)	617 (6.55)	142 (1.51)
Female	9076 (79.92)	3722 (29.08)	10511 (82.13)	2287 (17.87)	6904 (53.95)	3938 (30.77)	1956 (15.28)	12261 (95.80)	433 (3.38)	104 (0.81)
**Marital status**										
Single	2218 (68.23)	1033 (31.77)	2572 (79.11)	679 (20.89)	1575 (48.45)	1062 (32.67)	614 (18.89)	3064 (94.25)	130 (4.00)	57 (1.75)
Married	12180 (66.43)	6155 (33.57)	14528 (79.24)	3807 (20.76)	9534 (52.00)	5892 (32.14)	2909 (15.87)	17271 (94.20)	888 (4.84)	176 (0.96)
Divorced or widowed	379 (60.45)	248 (39.55)	480 (76.56)	147 (23.44)	326 (51.99)	196 (31.26)	105 (16.75)	582 (92.82)	32 (5.10)	13 (2.07)
**Children**										
None	4023 (68.98)	1809 (31.02)	4651 (79.75)	1181 (20.25)	2915 (49.98)	1876 (32.17)	1041 (17.85)	5523 (94.70)	234 (1.01)	75 (1.29)
One	8390 (65.34)	4450 (34.66)	10084 (78.54)	2756 (21.46)	6675 (51.99)	4109 (32.00)	2056 (16.01)	12069 (94.00)	640 (4.98)	131 (1.02)
More than one	2364 (66.76)	1177 (33.24)	2845 (80.34)	696 (19.66)	1845 (52.10)	1165 (32.90)	531 (15.00)	3325 (93.90)	176 (4.97)	40 (1.13)
**Educational level**										
Bachelor degree or below	3592 (63.50)	2065 (36.50)	4330 (76.54)	1327 (23.46)	2953 (52.20)	1715 (30.32)	989 (17.48)	5255 (92.89)	289 (5.11)	113 (2.00)
Master’s degree	7056 (67.41)	3411 (32.59)	8352 (79.79)	2115 (20.21)	5320 (50.83)	3393 (32.42)	1754 (16.76)	9878 (94.37)	494 (4.72)	95 (0.91)
Doctorate degree	4129 (67.81)	1960 (32.19)	4898 (80.44)	1191 (19.56)	3162 (51.93)	2042 (33.54)	885 (14.53)	5784 (94.99)	267 (4.38)	38 (0.62)
**Department**										
Internal medicine	4626 (70.02)	1981 (29.98)	5252 (79.49)	1355 (20.51)	3289 (49.78)	2236 (33.84)	1082 (16.38)	6224 (94.20)	310 (4.69)	73 (1.10)
Surgery	4752 (65.05)	2553 (34.95)	5750 (78.71)	1555 (21.29)	3942 (53.96)	2278 (31.18)	1085 (14.85)	6868 (94.02)	360 (4.93)	77 (1.05)
Ob/Gyn	1966 (63.28)	1141 (36.72)	2479 (79.79)	628 (20.21)	1619 (52.11)	979 (31.51)	509 (16.38)	2977 (95.82)	114 (3.67)	16 (0.51)
Pediatrics	1339 (59.88)	897 (40.12)	1663 (74.37)	573 (25.63)	890 (39.80)	796 (35.60)	550 (24.60)	2041 (91.28)	142 (6.35)	53 (2.37)
Emergency	746 (72.36)	285 (27.64)	837 (81.18)	194 (18.82)	602 (58.39)	284 (27.55)	145 (14.06)	972 (94.28)	47 (4.56)	12 (1.16)
Others	1348 (69.95)	579 (30.05)	1599 (82.98)	328 (17.02)	1093 (56.72)	577 (29.94)	257 (13.34)	1835 (95.23)	77 (4.00)	15 (0.78)
**Hospital type**										
General hospitals	5779 (64.93)	3121 (35.07)	6867 (77.16)	2033 (22.84)	4202 (47.21)	3083 (34.64)	1615 (18.15)	8265 (92.87)	521 (5.85)	114 (1.28)
TCM general hospital	4101 (66.94)	2025 (33.06)	4826 (78.78)	1300 (21.22)	3289 (53.69)	1879 (30.67)	958 (15.64)	5770 (94.19)	283 (4.62)	73 (1.19)
Specialty hospitals	4897 (68.14)	2290 (31.86)	5887 (81.91)	1300 (18.09)	3944 (54.88)	2188 (30.44)	1055 (14.68)	6882 (95.76)	246 (3.42)	59 (0.82)
**Location**										
East China	6500 (67.82)	3084 (32.18)	7777 (81.15)	1807 (18.85)	4985 (52.01)	3133 (32.69)	1466 (15.30)	9146 (95.43)	362 (3.78)	76 (0.79)
Central China	3524 (66.89)	1744 (33.11)	4099 (77.81)	1169 (22.19)	2757 (52.33)	1659 (31.49)	852 (16.17)	4885 (92.73)	326 (6.19)	57 (1.08)
West China	4753 (64.57)	2608 (35.43)	5704 (77.49)	1657 (22.51)	3693 (50.17)	2358 (32.03)	1310 (17.80)	6886 (93.55)	362 (4.92)	113 (1.54)

*All *P* < 0.001, except *P* for number of children to verbal violence (*P* = 0.003), and marital status to medical disturbances (*P* = 0.267).*

Among them, 33.48% reported medical disputes in the previous year. Higher rates of medical disputes were seen among physicians who were male, divorced or widowed, had one child, from a lower educational background, in pediatric departments, in general hospitals, and in West China. The logistic analysis showed that physicians who were older, working in obstetrics-gynecology (Ob/Gyn) and pediatrics departments, longer working hours/day, in West China were significantly more likely to experience (Supplementary Table 1).

One fifth (20.86%) of physicians reported experiencing medical disturbances, and male physicians reported a higher rate (24.92%) than female physicians (17.87%, *P* < 0.001). The overall trend of the subgroup analysis regarding medical disturbances was similar to that of medical disputes. Namely, the rate was higher in those who were divorced or widowed, had one child, had a lower educational background, and was in pediatric departments, general hospitals, and in West China. The logistic analysis showed that physicians who were older, working in Ob/Gyn and pediatrics departments, longer working hours/day, in Central China and West China were significantly more likely to experience medical disturbances (Supplementary Table 1).

Instances of verbal violence were also collected in the surveyed sample. In total, 48.52% reported experiencing verbal violence from patients and/or patients’ family members in the previous year (32.19% a few times a year, 16.33% a few times per month or more). The logistic analysis showed that physicians who were working in internal medicine, Ob/Gyn and pediatrics departments, working longer hours/day were more likely to experience verbal violence (Supplementary Table 1).

In total, 5.84% of physicians experienced physical violence in the previous year (4.73% a few times a year, 1.11% a few times per month or more). The logistic analysis showed that physicians who were older, working in pediatrics departments, working longer hours/day, in Central China and West China were more likely to experience physical violence (Supplementary Table 1).

### Factors Associated With Burnout

We performed logistic analyses to identify factors associating with overall burnout (Table 3). In the unadjusted model, which excluded the mistreatment factors, physicians who were female (odds ratio[OR], 0.91; 95% confidence interval [CI], 0.86–0.97), the elder (OR, 0.97; 95% CI, 0.97–0.98), with a doctoral degree (reference group: physicians with a college degree or below, OR, 0.81; 95% CI, 0.74–0.88), working in TCM general hospitals and specialty hospitals (reference: working in general hospitals, OR, 0.85; 95% CI, 0.79–0.91), were less likely to experience overall burnout. While those who were divorced or widowed (reference: single, OR 1.28; 95% CI, 1.05–1.57), in internal medicine and pediatric departments (OR, with surgery departments as a reference, 1.16, 1.35; 95% CI, 1.08–1.26, 1.21–1.50, respectively), longer working hours per day (OR, 1.17; 95% CI, 1.15–1.19), were more likely to experience overall burnout.

When mistreatment factors were included, the association with sex and pediatric departments was no longer significant. The regional difference remained significant, as physicians in Central China and West China (OR, with East China as a reference, 0.92; 95% CI, 0.85–0.99, respectively) were less likely to have overall burnout.

Compared with the group that reported no verbal violence, physicians who reported more frequent verbal violence were more likely to have overall burnout. A similar trend was found with physical violence (Table 3).

## Discussion

This study was the first nationwide survey on physician burnout in China and associated medical disputes, purposeful disturbances, violence among a large, nationally representative sample in China. By surveying physicians in tertiary public hospitals and obtaining a very high response rate, we comprehensively assessed the extent of burnout in China and its relationships to medical disputes, disturbances verbal and physical violence against physicians. We found that indeed the experience of mistreatment was significantly associated with physician burnout.

We found that the overall burnout rate in physicians in China is very close to the rate in Canadian pediatricians (34.1%) (Bennett et al., 2005), but lower than findings from other studies (West et al., 2016, 2018; The Lancet, 2019; Yates, 2020). For example, this percentage is lower than that of United States neurologists (60.1%) (Busis et al., 2017), United States oncologists (44.7%) (Shanafelt et al., 2014), United States surgeons (40%) (Shanafelt et al., 2009), French intensivists (46.5%) (Embriaco et al., 2007), Chinese neurologists (53.2%) (Zhou et al., 2017), and Chinese oncologists (51.0%) (Ma, 2017). Our findings are also lower than the findings of another online large sample survey (*N* > 15000, 29 specialties) in the United States, which found that 44% of physicians reported burnout (Lesile Kane, 2019). There are also some similarities: for example, the United States survey also found that female physicians, those working in internal medicine or Ob/Gyn departments, and those working longer hours were more likely to report burnout. The lower rate of burnout among Chinese physicians in this study, compared to their international counterparts, may be due to the overall high level of tolerance for workload in the continuously over-crowded hospitals, large and inclusive sample, very high response rate (>90%), and the timing of study (mid to late March) when physicians’ workload is traditionally lower in China (Zhang et al., 2018).

We found that one-third of physicians experienced medical disputes in the previous year in tertiary public hospitals. This high rate is very concerning. In recent years, the rate of medical disputes has increased rapidly in China, with the growth rate ranging from 11–24.5%, and the overall rate in this survey is even higher than all existing reports (Chen et al., 2008; Wu, 2015; Yu et al., 2018). The rate of medical disputes experienced by physicians varies from study to study. For example, Fan et al. (2012) found that there were 2.2 medical disputes per 100 physicians in Beijing and 1.32 per 100 physicians in Shanxi province, and both were lower than that in England and Germany, which were at 5.96 and 2.45%, respectively (Fan et al., 2012). Lu et al. (2015) showed that the rate of medical disputes was 5.89 per 100 physicians in 2010–2013. As many medical disputes are resolved by physicians and patients themselves without hospital management’s involvement, the data on the hospital-level is less than what was reported by physicians. Meanwhile, our findings showing that the physicians in the pediatrics, Ob/Gyn, and surgery departments reporting the highest rates of medical disputes is in line with several previous studies (Zhang and Zhao, 2014; Lin and Li, 2015; Wang et al., 2015).

In this study, 20.86% of physicians reported experiencing medical disturbances in the previous year. This alarmingly high rate of medical disturbances should serve as a warning, and it suggests up to a fifth of physicians in tertiary public hospitals were exposed to potential severe threats and safety risks at work. In recent years, the occurrence of medical disturbances has also sharply increased in China (The Lancet, 2020). According to a survey conducted by the Chinese Medical Doctors Association, within a sample of 48 hospitals, 47 hospitals (97.62%) had suffered from significant medical disturbances in 2006 (Chinese Medical Doctor Association, 2007). Our survey was the first to report national data of medical disturbances on an individual level among physicians.

According to our survey, the prevalence of frequent verbal violence and physical violence from patients or their family members was in line with other studies in China (Guo et al., 2019; Hu et al., 2019). In the past few years, some cases involving violence against physicians, especially those involving death or severe injury, have caused considerable public outrage. As an effort to curb such crimes, a law was recently hurriedly passed by the People’s Congress of China (Li and Gao, 2019).

Although medical disputes and workplace violence against doctors happen in almost all cultures, the overall situation has worsened in China’s past few decades (The Lancet, 2020). There, the patient-doctor relationship has become more contentious, and doctors are less respected. It has even affected the career trends among high school graduates, making them less likely to choose medicine as their career (Ma, 2017; Ou et al., 2019).

Many related factors, including cultural, institutional, and individual aspects, had been studied (Lin and Li, 2015; Lu et al., 2015; Amirthalingam, 2017; Reddy et al., 2019; Zhou and Hao, 2019). Concerning China’s situation, a few factors have been frequently discussed and considered to contribute to the rapid increase in these events. The proportion of the Chinese government’s health expenditure in the total national health expenditure is low, which was 58.0% in 2016, compared with the global average level of 74.3% (The World Bank, 2019). Meanwhile, the proportion of government input to the total income of public hospitals has been below 10% in recent years, which is one-third of the global average level (around 30%) (Hu and Zhu, 2019). On the social level, there is a conflict between the huge demand for health services and the limited rate of resource growth, especially in rural areas (Niu et al., 2016; Zeng et al., 2018). In the meantime, the cost of health care has increased rapidly, and many patients have unrealistic expectations (Zhao and Qiao, 2013; Wang et al., 2019). Professionally, many doctors in China lack the necessary training to be effective communicators and identify and deescalate conflicts (Wei et al., 2015). Hesketh et al. (2003) suggested that the “broken windows” theory might be a useful tool to conceptualize why workplace violence occurs and proposed proactiveness in dealing with minor forms of abuse or violence as early as possible.

In this study, mistreatment, more than any other individual or hospital characteristics, was significantly associated with overall burnout, consistent with the findings reported in a sample of surgery residents in the United States (Hu et al., 2019). This may be due to the negative psychological effects caused by mistreatment; meanwhile, the burnout physicians are also more likely to encounter mistreatment. The psychological status or the personality of a physician may be a core factor. Because the literature examining the association between mistreatment and physicians’ burnout is scarce, more studies are needed, especially studies examining pathways and mechanisms, effective interventions, or physicians’ recognition status.

Policymakers and hospital administrators need to be aware of these crucial issues, and urgent actions need to be taken, including increasing the healthcare budget, strengthening legal actions to protect physicians’ safety, hiring security guards in high-risk areas, recruiting more social workers, and improving staff training for effective communication and conflict resolution. A safer healthcare environment would improve physicians’ well-being and improve healthcare quality for patients.

Several limitations of this study should be pointed out. First, as in all cross-sectional studies, the causal relationship of different factors cannot be established. Second, our study did not include some other potentially influential factors. For example, personalities have been reported to be associated with burnout (Bughi et al., 2017; van der Wal et al., 2018), but we did not have data to measure it. Other potentially relevant factors, such as income level, staff and administrative support, and the use of electronic medical records (Fred and Scheid, 2018), have not been included in the survey. Third, we did not include any questions about sexual harassment, which is emerging as an essential aspect of the work environment. Fourth, the sample hospitals are tertiary public hospitals, not including primary medical units or other type hospitals, so the results may not be generalizable to all hospitals. Finally, we only focused on violence from patients and patients’ families, and we did not ask questions about violence from the staff. Studies show that violence from the staff was also common and as harmful as violence from patients (Abed et al., 2016; Moylan, 2017; Hu et al., 2019).

## Conclusion

In conclusion, we found that burnout, experiences of medical disputes, purposeful disturbances, and physical and verbal violence were common among Chinese physicians. These experiences were significantly associated with overall burnout. Our results provided some insights into why and how a safer, more sustainable healthcare system might be established.

## Data Availability Statement

All datasets presented in this study are available upon request.

## Ethics Statement

The protocol of this study was approved by the Ethics Committee (IEC) of the Emergency General Hospital in Beijing, China. The patients/participants provided their written informed consent to participate in this study.

## Author Contributions

YW, FJ, JM, Y-LT, MW, and YL: conceptualization. YW: data curation and investigation. FJ: data analysis and writing an original draft. YL: funding acquisition. FJ and Y-LT: methodology. FJ, JM, Y-LT, and YL: writing revision and editing. All authors contributed to the article and approved the submitted version.

## Conflict of Interest

The authors declare that the research was conducted in the absence of any commercial or financial relationships that could be construed as a potential conflict of interest.
