# Supplementary material for: Experience of Medical Disputes, Medical Disturbances, Verbal and Physical Violence, and Burnout Among Physicians in China
Source: Front Psychol. 2021 Jan 29;11:556517. doi: 10.3389/fpsyg.2020.556517 (PMC7878671; doi:10.3389/fpsyg.2020.556517)
Supplement: Supplementary file 1 [file Table_1.doc]

S1 Factors associated with Medical Disputes, Medical Disturbance, Verbal and Physical Violence

|  | | Medical disputes | | |  | Medical disturbance | | |  | Verbal violence | | |  | | Physical violence | | | |
| --- | --- | --- | --- | --- | --- | --- | --- | --- | --- | --- | --- | --- | --- | --- | --- | --- | --- | --- |
|  |  | odds ratio | 95% CI (Lower) | 95% CI (Upper) |  | odds ratio | 95% CI (Lower) | 95% CI (Upper) |  | odds ratio | 95% CI (Lower) | 95% CI (Upper) | |  | | odds ratio | 95% CI (Lower) | 95% CI (Upper) |
| Sex (reference: male) | |  |  |  |  |  |  |  |  |  |  |  | |  | |  |  |  |
|  | Female | 0.62 | 0.58 | 0.66 |  | 0.66 | 0.61 | 0.71 |  | 0.76 | 0.71 | 0.80 | |  | | 0.52 | 0.46 | 0.59 |
| Age | | 1.02 | 1.02 | 1.02 |  | 1.02 | 1.01 | 1.02 |  | 1.00 | 0.99 | 1.00 | |  | | 1.02 | 1.01 | 1.03 |
| Marriage status (reference: single) | |  |  |  |  |  |  |  |  |  |  |  | |  | |  |  |  |
|  | Married | 0.87 | 0.78 | 0.97 |  | 0.88 | 0.78 | 1.01 |  | 0.88 | 0.79 | 0.97 | |  | | 0.79 | 0.62 | 0.99 |
|  | Divorced or widowed | 1.14 | 0.94 | 1.38 |  | 1.03 | 0.83 | 1.29 |  | 0.96 | 0.79 | 1.16 | |  | | 1.03 | 0.70 | 1.50 |
| Children (reference: no children) | |  |  |  |  |  |  |  |  |  |  |  | |  | |  |  |  |
|  | One | 1.05 | 0.95 | 1.16 |  | 0.99 | 0.89 | 1.11 |  | 1.07 | 0.98 | 1.17 | |  | | 1.09 | 0.89 | 1.33 |
|  | More than one | 1.00 | 0.90 | 1.12 |  | 0.90 | 0.78 | 1.02 |  | 1.03 | 0.92 | 1.14 | |  | | 1.11 | 0.88 | 1.40 |
| Educational level (reference: college or below) | |  |  |  |  |  |  |  |  |  |  |  | |  | |  |  |  |
|  | Master’s degree | 0.92 | 0.86 | 0.99 |  | 0.86 | 0.79 | 0.94 |  | 1.00 | 0.93 | 1.07 | |  | | 0.81 | 0.71 | 0.94 |
|  | Doctorate degree | 0.83 | 0.77 | 0.91 |  | 0.77 | 0.70 | 0.85 |  | 0.94 | 0.86 | 1.01 | |  | | 0.63 | 0.54 | 0.75 |
| Department (reference: surgery) | |  |  |  |  |  |  |  |  |  |  |  | |  | |  |  |  |
|  | internal medicine | 0.88 | 0.82 | 0.95 |  | 1.03 | 0.94 | 1.12 |  | 1.25 | 1.16 | 1.34 | |  | | 1.11 | 0.95 | 1.28 |
|  | Ob/Gyn | 1.44 | 1.30 | 1.59 |  | 1.23 | 1.09 | 1.38 |  | 1.31 | 1.20 | 1.44 | |  | | 1.06 | 0.84 | 1.32 |
|  | Pediatrics | 1.45 | 1.31 | 1.60 |  | 1.44 | 1.28 | 1.61 |  | 2.01 | 1.82 | 2.22 | |  | | 1.78 | 1.48 | 2.15 |
|  | Emergency | 0.80 | 0.69 | 0.93 |  | 0.95 | 0.80 | 1.13 |  | 0.98 | 0.86 | 1.13 | |  | | 1.14 | 0.86 | 1.53 |
|  | Others | 0.86 | 0.77 | 0.97 |  | 0.80 | 0.70 | 0.92 |  | 0.97 | 0.88 | 1.08 | |  | | 0.86 | 0.68 | 1.09 |
| Working Hours/day | | 1.11 | 1.10 | 1.13 |  | 1.12 | 1.10 | 1.14 |  | 1.15 | 1.13 | 1.17 | |  | | 1.14 | 1.12 | 1.17 |
| Hospital type (reference: general hospitals) | |  |  |  |  |  |  |  |  |  |  |  | |  | |  |  |  |
|  | TCM general hospitals | 0.96 | 0.89 | 1.03 |  | 0.94 | 0.87 | 1.03 |  | 0.81 | 0.76 | 0.87 | |  | | 0.81 | 0.71 | 0.93 |
|  | Specialty hospitals | 0.83 | 0.77 | 0.90 |  | 0.77 | 0.70 | 0.84 |  | 0.75 | 0.70 | 0.81 | |  | | 0.64 | 0.55 | 0.75 |
| Location(reference: East China) | |  |  |  |  |  |  |  |  |  |  |  | |  | |  |  |  |
|  | Central China | 1.01 | 0.94 | 1.08 |  | 1.19 | 1.10 | 1.30 |  | 0.95 | 0.88 | 1.01 | |  | | 1.56 | 1.35 | 1.80 |
|  | West China | 1.10 | 1.03 | 1.18 |  | 1.16 | 1.07 | 1.26 |  | 1.01 | 0.95 | 1.08 | |  | | 1.27 | 1.11 | 1.47 |
